# Recommendations and Choices for BRCA Mutation Carriers at Risk for Ovarian Cancer: A Complicated Decision

**DOI:** 10.3390/cancers10020057

**Published:** 2018-02-21

**Authors:** Kelsey E. Lewis, Karen H. Lu, Amber M. Klimczak, Samuel C. Mok

**Affiliations:** 1 Department of Obstetrics & Gynecology, The University of Texas Medical Branch at Galveston, Galveston, TX 77555, USA; amklimcz@utmb.edu; 2 Department of Gynecologic Oncology and Reproductive Medicine, The University of Texas MD Anderson Cancer Center, Houston, TX 77030, USA; khlu@mdanderson.org (K.H.L.); scmok@mdanderson.org (S.C.M.); 3 The University of Texas Graduate School of Biomedical Sciences at Houston, Houston, TX 77030, USA

**Keywords:** BRCA, ovarian cancer, risk-reducing surgery, CA-125, screening guidelines

## Abstract

Current ovarian cancer screening guidelines in high-risk women vary according to different organizations. Risk reducing surgery remains the gold standard for definitive treatment in BRCA mutation carriers, but research advancements have created more short-term options for patients to consider. The decisions involved in how a woman manages her BRCA mutation status can cause a great deal of stress and worry due to the imperfect therapy options. The goal of this review was to critically analyze the screening recommendations and alternative options for high-risk ovarian cancer patients and evaluate how these discrepancies and choices affect a woman’s management decisions.

## 1. Introduction

More than 14,000 deaths from ovarian cancer are predicted in the year 2017 [1]. In the United States, ovarian cancer is responsible for more deaths than all other gynecologic cancers combined [2]. It has a low prevalence but high mortality rate, with nearly 70% of patients presenting with late-stage ovarian cancer. In women with BRCA1 or BRCA2 mutations, the incidence of developing cancer is much higher than the average-risk woman [3]. The ovarian cancer risk is about 39–46% for BRCA1 and 10–28% for BRCA2 by age 70 [4,5,6,7]. BRCA 1 and BRCA2 account for 15–22% of all high-grade serous ovarian cancer cases and 9–24% of all epithelial ovarian cancer cases [8,9,10]. The ovarian cancer associated with BRCA mutations is also more likely to be high-grade and endometrioid or serous subtype [11].

## 2. Recommendations for Ovarian Cancer Screening in High-Risk Women

There is no perfect screening method to detect ovarian cancer. Surveillance is especially critical for women with BRCA1 and BRCA2 mutations due to the increased risk of ovarian cancer. There is still no absolute consensus on screening frequency and method for these high-risk women. The current recommendations from the American College of Obstetricians and Gynecologists (ACOG) suggest that women at high risk may choose screening with CA-125 serum levels and transvaginal ultrasound (TVUS) starting at age 30–35, although screening is generally not recommended [12]. If a family member was diagnosed with ovarian cancer, then this screening should begin 5–10 years earlier than the family member’s age at the time of their diagnosis. ACOG also recommends risk reducing salpingo-oophorectomy (RRSO) after childbearing is completed or at 35–40 years of age for BRCA1 carriers or at 40–45 years of age for BRCA2 carriers. According to the group, patients should be informed about lack of evidence supporting routine screening and that routine screening does not reduce mortality. The Society of Gynecologic Oncology (SGO) agrees with the current ACOG endorsements [13,14]. The National Comprehensive Cancer Network (NCCN) in the US promotes RRSO in high-risk women, with the option of having TVUS and CA-125 screening, which is done on a case-by-case basis [15]. The United States Preventative Services Task Force (USPSTF) does not have formal recommendations on screening in high-risk women, citing the lack of evidence for intensive screening [16]. In the United Kingdom, the National Institute for Health and Care Excellence (NICE) does not endorse screening in this high-risk population, and it is therefore not funded by the National Health Service [15]. The screening recommendations are described in Table 1.

## 3. Screening Trials

The tumor antigen CA-125 and TVUS have remained the focus of many large ovarian cancer screening trials to date. Serum levels of the biomarker CA-125 can be monitored in known ovarian cancer patients to recognize recurrence or initiate suspicion if the level is greater than the designated cutoff value of 30–35 U/mL [17,18]. CA-125 has limited specificity, however, as a result of its fluctuation in many benign conditions; only about one-half of early-stage ovarian cancer patients will have elevated levels [19]. The use of TVUS also leads to increased false-positive screens from detection of benign adnexal masses that cannot be ruled out [20]. Major surgery is required to diagnose a mass as ovarian cancer due to the lack of an acceptable intermediate confirmatory test. Biopsy of an ovarian mass is not recommended due to the risk of spillage and seeding of malignant cells in the peritoneal cavity [21]. Intraoperative rupture of a malignant epithelial ovarian cancer has been shown to worsen survival of patients with stage I cancers [22]. Thus the morbidity of a major operation needed to diagnose, along with an overall low incidence of ovarian cancer, establishes the challenge of finding a cost-effective and readily-available screening test. It has been suggested that an effective screening test for ovarian cancer should have a specificity of greater than 99% and a sensitivity of >75% to reach the target PPV of at least 10% [13,23].

In 1992, the Prostate, Lung, Colorectal, and Ovarian Cancer Screening Trial (PLCOCS) was initiated with the primary objective of determining the effect of screening average-risk women with CA-125 and TVUS on mortality [24]. For the study, 34,253 women were randomized to the screening intervention group, and 34,304 to the usual care (no screening) group. The intervention group was offered annual screening with CA-125 for six years and TVUS for four years. A fixed CA-125 result of 35 U/mL or greater was classified as abnormal in this study. When the trial concluded, there had been 118 deaths in the intervention group and 100 deaths in the usual care group. They found an increased risk of complication from false-positive results with no reduction in ovarian cancer mortality [25,26,27]. Another randomized control trial that assessed the effect of screening on mortality was the UK Collaborative Trial of Ovarian Cancer Screening (UKCTOCS). Over 200,000 average-risk postmenopausal women were assigned to receive usual care, an annual TVUS, or an annual CA-125 screening with second-line TVUS. This study utilized the risk-of-ovarian-cancer algorithm, or ROCA. Instead of a fixed CA 125 level as employed in the PLCOCS, ROCA utilizes a woman’s established baseline CA 125 level and then evaluates for an increase or variation in level. This change point based on a woman’s age and baseline CA 125 is then used to calculate the individual risk of malignancy. The change in CA 125 profile may lead to an increase in screening sensitivity while maintaining a high specificity [28]. Enrollment for the UKCTOCS began in years 2001–2005 and the screening period was concluded in 2011. For primary invasive epithelial ovarian and tubal cancers, the sensitivity, specificity, and PPVs were 89.5, 99.8, and 35.1%, respectively, for MMS (multi-modal screening) and 75.0, 98.2, and 2.8%, respectively, for TVUS. There was a significant difference in specificity but not sensitivity between the two screening groups [29]. When the screening ended in 2011, the MMS group had a mortality reduction of 15% (*p* = 0.10) and the TVUS group 11% (*p* = 0.21) [30]. Also ending in 2011, a single-arm prospective study using ROCA to screen 4051 average-risk postmenopausal women was conducted by researchers in the US and compared to the UKCTOCS [23]. An annual CA-125 level placed patients into three categories based on the ROCA score: repeat CA-125 in a year (low risk), repeat CA-125 in three months (intermediate risk), or TVUS and referral to a gynecologic oncologist (high risk). The rate of referral to a three-month CA-125 was 5.8%, and less than 1% was placed in the high-risk category. The specificity was 99% and PPV was 40% compared to 35.1% in the UKCTOCS.

To assess women at high risk of ovarian cancer (>10%), the first large prospective trial of this population screened 3563 women in the United Kingdom Familial Ovarian Cancer Screening Study (UKFOCSS) [31]. Phase I involved annual CA-125 levels and a TVUS. A single cutoff value for CA-125 was set in premenopausal women as 35 IU/mL and in postmenopausal women as 30 IU/mL. The PPV of incident screening was 25.5%. Cancer detected in women who had not had screening the previous year was more likely to be > stage IIIc disease (*p* = 0.009). The median interval from detection screen to surgery was 79 days in the ovarian cancer patients. Protocol-driven follow-up time was implemented in Phase II of the UKFOCSS to decrease surgery delay. This portion of the study took place during the last two years. Screening in these women was increased to once every four months instead of annually. Only 42% of cancers detected were stage I/II, but 92% of all detected were able to be completely cytoreduced compared with 62% in Phase I [31].

In a large US trial published in 2017, data from the Cancer Genetics Network and Gynecological Oncology Group trials was used to screen 3692 high-risk women. CA-125 was measured every three months and assessed using ROCA [20]. If the new risk (of having ovarian cancer) was calculated at <1%, then a patient returned again in three months for CA-125. If significant increases in CA-125 above baseline placed a patient at intermediate risk (1–10%) then a TVUS was performed. High-risk (>10%) patients received a TVUS and referral to a gynecologic oncologist. The study had a specificity >90% and PPV of 4.6%. A total 50% of the ROCA-detected cases were caught before the standard cutoff value of 35 for CA-125. There was a promising 88% compliance rate in this high-frequency study. The screening trials are described in Table 2.

## 4. Risk-Reducing Surgical Options

Despite the developments in recent trials, there is still no effective ovarian cancer screening test for high-risk women. Therefore, RRSO remains the gold standard for women at high risk of developing ovarian cancer. ACOG and the SGO agree that RRSO in these women should be performed after completion of child bearing or by the age of 40 after discussing risks vs. benefits of surgery [12,13,14]. The risk by age 40 is about 3% and jumps to about 10% by the age of 50. It has been shown that RRSO can reduce the risk of ovarian cancer by 80% [32]. 

As a means to an alternative for patients not desiring surgery however, chemopreventive agents have been evaluated in high risk women. Chemoprevention has been defined as the inhibition of carcinogenesis using natural or synthetic agents [33,34]. Oral contraceptives may decrease ovarian cancer risk due to their inhibitory effects on ovulation or due to the apoptotic effect of progestins on the ovarian epithelium [34]. One of the largest case control studies to assess this effect of oral contraceptives found a reduced risk of ovarian cancer in carriers of *BRCA1* mutations (odds ratio 0.56 (95% CI 0.45–0.71); *p* < 0.0001) and carriers of *BRCA2* mutations (0.39 (95% CI 0.23–0.66); *p* = 0.0004) [35]. Some studies have also shown a slightly increased risk reduction of breast cancer with oral contraceptives in BRCA1 carriers [36]. A large meta-analysis did not find a statistically significant correlation between the increased breast cancer risk and oral contraceptive use in high risk patients [37]. There is currently not enough evidence to recommend the use of chemoprevention in place of risk reducing surgery in BRCA mutation carriers.

Despite risk reducing surgery prevailing as the gold standard for treatment, there are substantial risks to undergoing RRSO. If performed at the recommended age of 40, women will experience surgical menopause on average an entire decade earlier than women who retain their ovaries. Postmenopausal ovaries will continue to produce a low level of hormones, even in elderly women, that provides a protective effect. The dramatic drop in estrogen will cause vasomotor symptoms, loss of libido, and dyspareunia in 90% of premenopausal women [38]. In addition, it has been proven that the lack of estrogen is associated with increased risks of stroke, cardiovascular disease, osteoporosis, and neurological conditions, such as Parkinsonism and dementia [39,40,41,42]. Testosterone production also continues after menopause but is decreased by approximately 25% [43]. Testosterone has been shown to have an impact on sexual function and desire [44].

In light of these increased risks, research has been focusing on a less radical alternative to RRSO as the solution in high-risk women. The increase in RRSO procedures over the years and number of pathology specimens it has provided has led to improved knowledge of ovarian cancer development. The rate of RRSO specimens found to have an occult malignancy has been 3–8% [45,46]. The results from the Gynecologic Oncology Group (GOG) trial-0199 study that assessed the surgical intervention arm of a larger prospective screening study in high-risk women concluded that ovarian/tubal neoplasms occur in 2.6% of women undergoing RRSO [47], including 4.6% of BRCA1 carriers and 3.5% of BRCA2 carriers. A majority of these malignancies have been discovered in the fallopian tube and serous tubal intraepithelial carcinoma (STIC), leading to the hypothesis that the fimbriated end of the fallopian tube is the origin of most high-grade serous ovarian cancers [48,49]. In addition, it has been found that up to 96% of high-grade serous ovarian cancers have a TP53 loss, which is a feature of fallopian carcinogenesis, further supporting the theory [49,50,51]. The association between bilateral tubal ligation and a decrease in ovarian cancer has also already been established [52,53]. A recent meta-analysis demonstrated a reduction in ovarian cancer by 34% after tubal ligation, and the protective effect was confirmed up to 14 years after the surgery [54]. Another nested case-control study identified 194 serous epithelial ovarian cancer and peritoneal cancer cases and matched them with 388 controls. The cancer rate was decreased by 64% after tubal ligation compared to those with nonexcisional tubal sterilization [55]. A recent survey in Australia revealed that due to the increasing evidence supporting the link between ovarian cancer and fallopian tube origin, 70% of clinicians in Australia are now offering opportunistic bilateral salpingectomies to their low risk patients [56]. In 2017 ACOG also reaffirmed their recommendation for physicians to discuss bilateral salpingectomy in low risk women at time of hysterectomy to prevent ovarian cancer [57]. A large retrospective cohort study in British Columbia evaluated over 43,000 low risk women who underwent hysterectomy vs. hysterectomy with bilateral salpingectomy as well as women who underwent bilateral salpingectomy vs. tubal ligation. Operative time for bilateral salpingectomy with hysterectomy was on average 16 min longer by any approach, and in sterilization surgeries on average 10 min longer than tubal ligation alone [58]. There was no increase in length of hospital stay, hospital readmission, or blood transfusions. Based on these results the practice of opportunistic salpingectomy in low risk patients was deemed safe and very reasonable. The long term safety of bilateral salpingectomies is still being investigated. In a recent observational study, 79 low risk women that underwent a total laparoscopic hysterectomy with prophylactic bilateral salpingectomy were followed and no negative effects on ovarian function were observed three to five years after surgery [59].

With these research advancements and increasing implementation of bilateral salpingectomy in the low risk population, bilateral salpingectomy with ovarian retention is being considered by some as a temporary bridge in high-risk women. A radical fimbriectomy is a procedure involving removal of both fallopian tubes including the entire fimbriated end. This removal of the fimbrio–ovarian junction involving a small portion of the ovary also removes at-risk cancer precursors [60,61]. A bilateral salpingectomy with ovarian retention allows high-risk women who may have initially declined RRSO an alternative option that will delay menopause and preserve fertility, if desired, with the option of assisted reproductive technologies. Wide excision of fallopian tube tissue adjacent to the ovary has been shown to have no effect on ovarian reserve [62]. The surgery can be performed in most women with a simple laparoscopic technique that also allows for clinical inspection of the peritoneal cavity and ovaries. Moreover, the overall risk reduction of a radical fimbriectomy may be greater than expected due to the advanced stage at which most ovarian cancers are detected, which may be masking a true fallopian tube origin [63]. 

This lack of understanding about the true origin of ovarian cancers is also a potential problem with bilateral salpingectomy with ovarian retention if the fraction of true tubal origins is less than anticipated. Despite the “simple” label given to a laparoscopic procedure, the surgical morbidity is still of concern due to the need for a second surgery to remove the ovaries at a later date. The effect of breast cancer risk reduction is also diminished when the ovaries are not removed prior to menopause in BRCA carriers [64]. Lastly, this procedure may delay or prevent some high-risk women from ever undergoing complete RRSO, which still remains the gold standard of treatment. 

## 5. Psychosocial Stress of BRCA Mutation Carriers

High-risk women are thus left with the choice of imperfect screening methods or invasive and life-altering surgery. An individual woman’s perception of risk versus actual risk of developing cancer is based on complex dynamics and influences. It has been shown in the study of other diseases that affective states such as worry, fear, and emotions tend to emerge as stronger predictors of behavior than cognitive risk [65]. Cognitive risk is created based on a woman’s existing knowledge and the objective findings given to her.

Due to the nature of BRCA mutation carriers’ increased frequency of screening, it was predicted that the likelihood of experiencing a false-positive test result may lead to increased perception of elevated cancer risk [66]. In this study done at the National Cancer Institute (NCI), women who experienced false-positive test results were found to experience transient emotional reactions that usually returned to baseline by one year post testing. Worry, however, which was measured using Lerman’s breast cancer worry scale, was shown to be a stronger predictor of risk perception than cognitive cancer worry. Those above the median for ovarian cancer worry had more than six times the odds of undergoing RRSO (OR = 6.15). 

A systematic review that analyzed the psychological impact in BRCA mutation carriers also found that the anxiety and depression associated with the distress experienced in the first 12 months after genetic testing generally does not linger in the intermediate and long term [67]. 

To assess effects of identified cancer burden, a large Gynecologic Oncology Group (GOG) trial evaluated 2287 questionnaires in high risk women that measured worry, perceived cancer risk, risks vs. benefits, and quality of life. The more invasive RRSO option, which 40% of the participants chose, was associated with higher perceived risk and worry [68]. Half of all the participants estimated their lifetime ovarian cancer risk to be >50%, which is greater than the actual risk. Another study performed by the NCI evaluated how young BRCA carriers (average age 29) viewed their risk perception [69]. How they interpret meaning of their diagnosis from family and doctors contributed greatly towards decision making. The nononcologic components of perceived risk regarding assumptions and effect on family and children played a greater role than anticipated. Many of these young BRCA carriers chose screening over RRSO. These studies reflect a continued goal to help patients make an informed evidence-based decision. 

In a large UK study, women who chose screening over RRSO will return to the surgical option many years after being diagnosed with BRCA status. This emphasized the need for close follow up, as decision making is often delayed in high-risk women. Postmenopausal women were 2.16 times more likely to choose RRSO [70]. The main factors affecting decision making were being postmenopausal, having a personal history of cancer, and having children. Another study that asked 21 high-risk women who discontinued routine screening and chose RRSO found that abnormal screening test results were the main deciding factor. The abnormal results prompted a discussion with the provider and a recalculation of risk, resulting in RRSO and supporting regular provider visits to revisit risk and options [71].

Investigators from MD Anderson reviewed 313 patient responses regarding satisfaction after RRSO and compared them to those who opted for periodic screening. Using the Satisfaction With Decision (SWD) scale, the median score was significantly higher in the RRSO group compared with the periodic screening group (*p* = 0.1) [72]. Lower satisfaction levels in women who chose periodic screening were associated with uncertainty and difficult decision making, perhaps due to the door left open for choice. The adverse effects of RRSO were surprisingly not associated with satisfaction level. 

To evaluate the need for social support and how it impacts decision making, a group from Norway enrolled BRCA mutation carriers in an educational support group [73]. It was found that having a patient representative who was able to lead discussion and answer questions from a personal standpoint improved patient knowledge and led to an increased feeling of power over the situation. New questions were generated, and the opportunity to share thoughts and emotions with other carriers provided an emotional release, which has been shown to provide better treatment outcomes [74]. 

## 6. Conclusions

The current screening guidelines generally do not endorse routine screening in high risk patients. Nonetheless, screening with CA 125 and TVUS for ovarian cancer in these patients may be done beginning around 30–35 years of age with plans for eventual RRSO. Bilateral salpingectomy with ovarian retention is another potential option for interval treatment management in BRCA mutation carriers. The individualization of therapy options leads to a difficult decision compounded by worry and stress that may not be fully appreciated by practitioners. BRCA mutation carriers should be encouraged to maintain regular visits with their physicians to discuss current guidelines and their changing personal goals. The cancer risk should be discussed again at each encounter, and all patients should be given the option of incorporating a support group into the treatment plan to minimize misperceptions and address concerns that affect decision making.

## Figures and Tables

**Table 1:** Screening recommendations (condensed).

Organization	Routine Screening	Screening Method	Frequency of Screening	Surgical Treatment
**American College of Obstetricians and Gynecologists** **(ACOG)**	Routine screening generally not recommended. Short term surveillance until RRSO is reasonable	Transvaginal ultrasound (TVUS) or CA 125	Starting at age 30–35, or 5–10 years earlier than family member’s age at time of diagnosis	Risk reducing salpingo-oophorectomy (RRSO) at age 35–40 for BRCA1, at age 40–45 for BRCA2
**Society of Gynecologic Oncology** **(SGO)**	Routine screening generally not recommended. Short term surveillance until RRSO is reasonable	TVUS or CA 125	Starting at age 30–35, or 5–10 years earlier than family member’s age at time of diagnosis	RRSO at age 35–40 for BRCA1, at age 40–45 for BRCA2
**National Comprehensive Cancer Network** **(NCCN)**	Routine screening not recommended	TVUS or CA 125	Case-by case basis beginning at age 30–35	RRSO at age 35–40 or at completion of childbearing. May delay until age 40–45 for BRCA2 if patient has had a bilateral mastectomy
**US Preventative Services Task Force** **(USPSTF)**	No recommendations in high risk population			

**Table 2:** Screening trials (condensed).

	PLCOCS [24]	UKCTOCS Multimodal [30]	UKCTOCS Ultrasound [30]	Lu and Colleagues [23]	UKFOCSS (Phase I) [31]	Skates and Colleagues [20]
**Objective**	Mortality of ovarian cancer screening with CA-125 and TVUS	Assess the effect of ovarian cancer screening on mortality	Assess the effect of ovarian cancer screening on mortality	2-stage screening that evaluates change in CA-125 over time to estimate risk of ovarian cancer	Assess annual TVUS and CA-125 for high-risk women	Assess increased frequency screening using CA-125 determined by ROCA in high-risk women
**Study design**	RCT	RCT	RCT	Single-arm, prospective study	Prospective study	Prospective study
**Screening strategy**	Annual screening with CA-125 for 6 years and TVUS for 4 years	Annual CA-125 using ROCA→TVUS as second line	Annual screening using TVUS alone	ROCA→annual CA-125 or 3 month CA-125 or TVUS/gyn onc	Annual TVUS and CA-125	Q3 month CA-125 using ROCA→TVUS
**Number screened**	34,253	50,078	48,230	4051	3563	3692
**Number of surgeries**	1771	97	845	10	637	195
**Number of invasive ovarian cancers**	212	42	45	4	37	9
**Number of borderline or low malignant potential tumors**		8	20	2		1
**PPV**		35%	2.8%	40%	25.5%	4.6%
**Specificity**		99.8%	98.2%	99.9%	98.9%	92%
